# FoamPi: An open-source raspberry Pi based apparatus for monitoring polyurethane foam reactions

**DOI:** 10.1016/j.ohx.2022.e00365

**Published:** 2022-09-28

**Authors:** Harry C. Wright, Duncan D. Cameron, Anthony J. Ryan

**Affiliations:** a Department of Chemistry, the University of Sheffield, Sheffield S3 7HF, UK; b Plants, Photosynthesis and Soil, School of Biosciences, the University of Sheffield, Sheffield S10 2TN, UK

**Keywords:** Polyurethane, Kinetics, Raspberry pi, Foam, Adiabatic temperature rise

## Abstract

Adiabatic temperature rise is an important method for determining isocyanate conversion in polyurethane foam reactions as well as many other exothermic chemical reactions. Adiabatic temperature rise can be used in conjunction with change in height and mass measurements to gain understanding into the blowing and gelling reactions that occur during polyurethane foaming as well as give important information on cell morphology. FoamPi is an open-source Raspberry Pi device for monitoring polyurethane foaming reactions. The device effectively monitors temperature rise, change in foam height as well as changes in the mass during the reaction. Three Python scripts are also presented. The first logs raw data during the reaction. The second corrects temperature data such that it can be used in adiabatic temperature rise reactions for calculating isocyanate conversion; additionally this script reduces noise in all the data and removes erroneous readings. The final script extracts important information from the corrected data such as maximum temperature change and maximum height change as well as the time to reach these points. Commercial examples of such equipment exist however the price (>£10000) of these equipment make these systems inaccessible for many research laboratories. The FoamPi build presented is inexpensive (£350) and test examples are shown here to indicate the reproducibility of results as well as precision of the FoamPi.

## Graphical abstract

**Figure:**
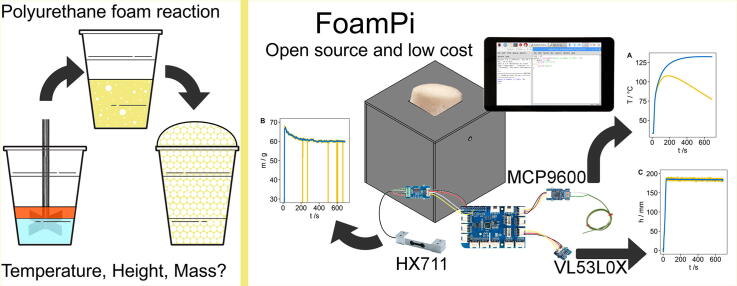

Specifications table.

**Table:** 

Hardware name	FoamPi
Subject area	●• Engineering and materials science ● Polymer Science
Hardware type	● Lab scale system for measuring polyurethane foam reactions.
Closest commercial analog	Foamat 285
Open source license	Creative Commons Attribution-ShareAlike 4.0 International License (CC BY-SA 4.0)
Cost of hardware	£350.93
Source file repository	https://doi.org/10.17605/OSF.IO/U3295

## Hardware in context

Temperature rise has been used extensively as a method to determine the reaction kinetics of polyurethane foams and other chemistries. This method takes advantage of the highly exothermic reactions that occur during the formation of polyurethane foams as well as the low thermal conductivity of the foams. Initially adiabatic temperature rise was shown to be an effective method for monitoring kinetics for fast cure polyurethane reactions as they are easy to monitor with non-specialised equipment as reactions are fast and polyurethanes have low thermal conductivity, meaning that heat loss from the system is low [1]. Additionally with a heat loss correction, adiabatic temperature rise can be used for polyurethanes with longer reaction times as well [2] and this heat loss correction also helps account for any heat loss from open systems or non-insulated reaction vessels. Isocyanate conversion calculated from adiabatic temperature rise data has been closely correlated to isocyanate conversion from infrared spectroscopy data, verifying the use of this approach for determining isocyanate conversion [3]. Adiabatic temperature rise has also been used to model the blowing and gelling reactions for water blown polyurethane foams. Models fitting first order kinetics to the blowing reaction were in good agreement with the experimental data up to a temperature of 140 °C [4]. Further modelling work using temperature rise has successfully modelled rigid polyurethane foams temperature rise, including the estimation of important kinetic properties such as pre-exponential factors, Arrhenius activation energy and heat of reactions [5]. Adiabatic temperature rise has also proved useful in comparing and understanding reaction rates of soy bean based polyols against conventional polyols [6]. These studies support the use of adiabatic temperature rise for monitoring polyurethane foam kinetics and isocyanate conversion. In addition to polyurethane foams there is a broader application for capturing adiabatic temperature rise for understanding the kinetics of many chemical reactions with a Web of Science search of “adiabatic temperature rise” returning 30 – 50 publications a year for the last 5 years in many areas including phenolics, casting, resins, concrete etc.

The production of CO_2_ during the blowing reaction, causes an increase in volume of polyurethane foam (PUF) and has been used to monitor the progress of the blowing reaction. Van Thuyne and Zeegers [7] studied flexible PUF reactions and tracked the change in height of a foam using a light sensor which followed the foam height. They found this method well suited for flexible foam, with a large advantage being that the instrumentation did not contact the foam, allowing free foam rise. Baser et al., [4] recorded the change in foam height using clear cylindrical reaction vessels and a video camera. They were able to model water blown polyurethane foams as having a first order kinetics with regard to water concentration. It would therefore be beneficial to measure foam rise height, and an additional requirement would be doing so without disturbing the foam surface.

When cell walls rupture during the PUF reaction, CO_2_ can escape and this causes a measureable mass loss. The extent of cell opening plays an important role in determining the final physical properties of PUF and having a mass loss curve during the reaction can give important insights into cell morphology. Furthermore, Knowledge of the mass of the reactants is also important for ensuring reproducible experiments. Shen *et al.,* [8] modelled PUF box foam density using height and mass loss data, for a low boiling point blowing agent and water. The mass loss during mixing and degassing explained the inefficiencies in the low boiling point blowing agent. It would therefore be beneficial and complementary to the temperature and height data to record mass loss data.

There is a commercially available apparatus that can monitor polyurethane reactions, called the FOAMAT (Format Messtechnik GmbH, Karlruhe, Germany) which captures the temperature change, change in height and mass loss (by measuring mass loss of reagents that remains in the mixing couple as opposed to those poured into the apparatus). In addition to these properties the FOAMAT is also able to measure the rise pressure (used to determine blow off point for flexible foams and reveals an objective gel point) and the dielectric polarization (used to determine the time of completion of the reaction). The FOAMAT has been used as an apparatus to model kinetic parameters and viscosity of polyurethane foams [9], used to determine the effect of biobased polyols on polyurethane foam reaction profiles [10], [11] as well as determine reaction profiles of biobased furanic foams [12]. Foam producers also use the FOAMAT apparatus extensively in industry. However the cost of the apparatus can be prohibitive for research laboratories and the FoamPi was developed with this in mind.

## Hardware description

**Fig. 1:**
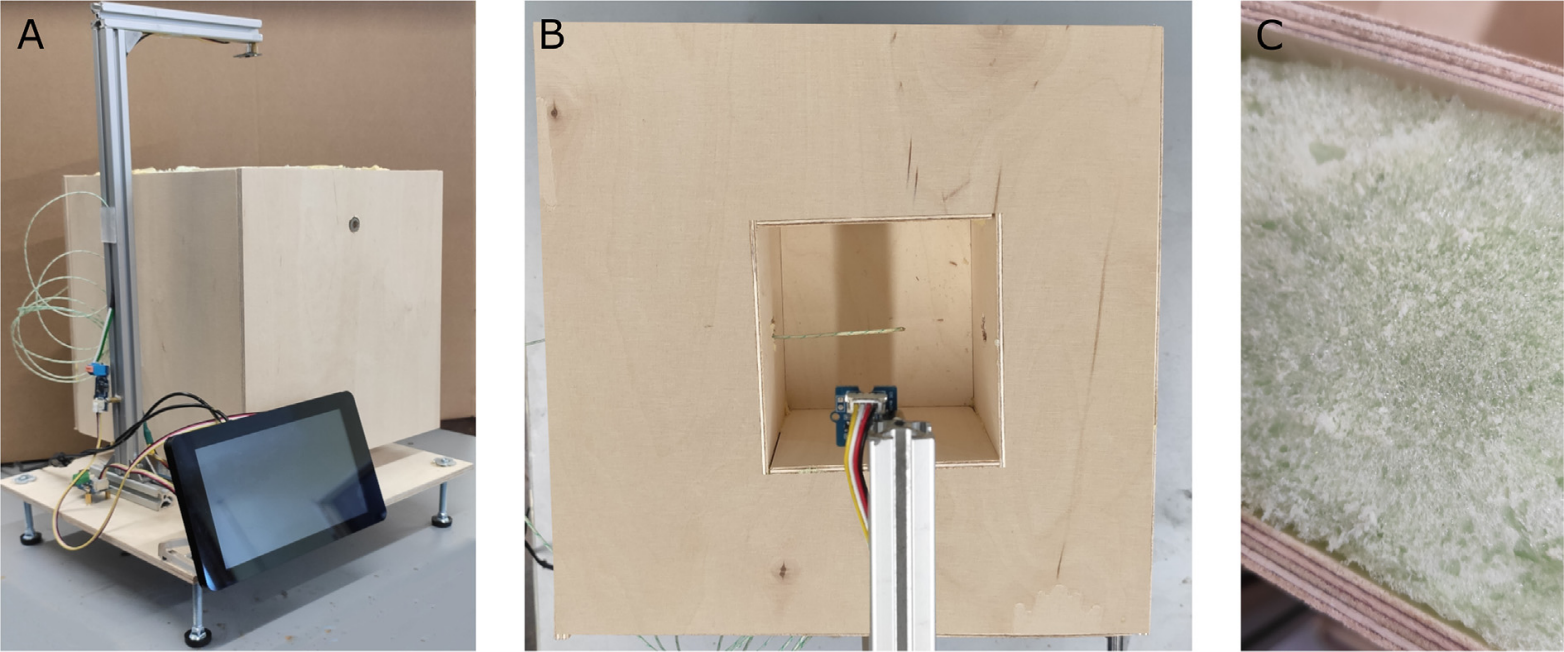
(A) The FoamPI, a low cost apparatus for monitoring polyurethane foam reactions, (B) top view of the box in box reaction vessel with time of flight sensor and thermocouple in view and (C) the layer of expanded foam insulation between the two boxes.

Commercial equipment for monitoring polyurethane reactions is expensive and the objective of this work is to design low cost apparatus for accurately monitoring polyurethane foaming reactions. The FoamPi consists of a Raspberry Pi 4 4 GB with a GrovePi + bridge to make the addition or removal of sensors easier and reduce the complexity of coding required to interpret the signals from sensors. Temperature is logged with a MCP9600 I2C board and k-type thermocouple. Height is logged using a VL53L0X time of flight (TOF) I2C sensor, a laser-ranging module that can measure distance accurately irrespective of the reflecting surface. Mass is logged using a HX711 ADC with a 0–3 kg load cell. All measurements are recorded through a python script and saved as a csv file. In addition to the electronics the FoamPi includes the reaction vessel, consisting of a 100 mm × 100 mm × 100 mm box housed in a larger box (250 mm × 250 mm × 250 mm) made from plywood with a layer of insulation between the two to reduce temperature loss from the reaction vessel. Fig. 1 shows the complete FoamPi as well as a top view with the box in box reaction vessel visible as well as the layer of expanded foam insulation to reduce heat loss from the system. The use of easily available sensors and code in conjunction with using simple wooden materials means that the FoamPi cost is kept low (∼£350).

A further benefit of the FoamPi is the additional python scripts that help with data correction and analysis, particularly with regards to temperature rise. This script corrects for temperature loss, and converts the raw temperature rise data into adiabatic temperature rise, which can be used for determining kinetic parameters or isocyanate conversion [1]. The mass and height data are also corrected by removing any erroneous data (such as the mixing cup moving in front of the TOF laser when pouring into the reaction vessel) and a 21 point moving average is applied to the data from each sensor to reduce noise. The second python script extracts important points from the corrected sensor data that can be useful for determining kinetic parameters, these include but are not limited to: maximum reaction temperature, maximum height, time of maximum temperature, time of maximum height and percentage sag.

**Figure A1:**
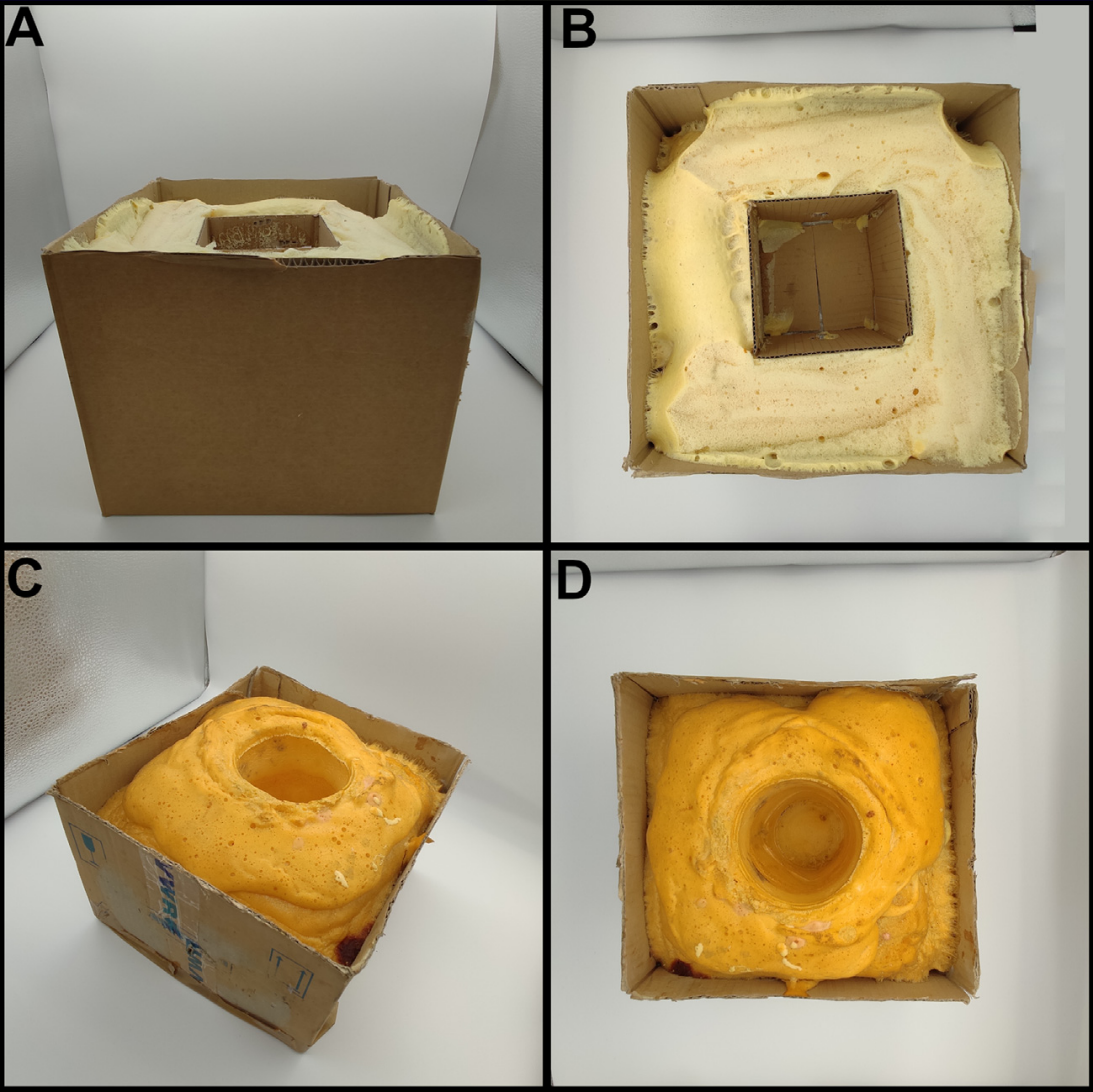

The cost of the FoamPi can be further reduced if the user is not interested in the mass change of the foam. Instead of the wooden box in box reaction vessel two cardboard boxes can be used instead. Additionally, for this reduced build the thermocouple breakout board as well as the TOF laser connect directly to the GPIO pins of the Raspberry Pi and therefore the GrovePi + bridge is not required. Furthermore, the touch screen can be removed and the RPi can be run headless with VNC software if further cost reductions are required. The total price of this simplified FoamPi is roughly £125. Two examples of these boxes are shown in the supplementary data Figure A1 (https://osf.io/9ugqz). These simple cardboard box-in-box reaction vessels were designed for completing a PUF reaction in either polypropylene cups or 100 mm cardboard boxes where mass is not required. They are insulated with 50 – 100 mm foam between the inner and outer box to reduce heat loss, although the use of the heat loss correction means this may not be required.

Although the FoamPi is designed for the monitoring of polyurethane foams it can be used for any reactions that require monitoring of temperature, height and mass or a subset of these. An example as described above could be the monitoring of furfural based foaming reactions [12]. A RPi with solely a thermocouple attached has also found much use in our laboratory as a cheap reliable logging thermocouple and has been used for several polymer synthesis reactions [13].

Uses of the FoamPi include:

• Determining urethane and blowing reaction kinetics of polyurethane foams.
• Comparing foaming reaction profiles of novel bio based polyols to industry standards.
• Determining activities of catalyst/surfactant polyurethane packages.
• Determining reaction kinetics of exothermic non-urethane foams (e.g. phenolic foams, polyisocyanurate foams, tannin based foams).
• Monitoring and logging of temperature of non-foaming chemical reactions.

## Design files summary

**Table:** 

**Design file name**	**File Name**	**File type**	**Open source license**	**Location of the file**
*Design File 1*	100mmBox_Bot	Cad File (.FCStd)	CC by 4.0	https://osf.io/dr2c3/
*Design File 2*	100mmBox_Front	Cad File (.FCStd)	CC by 4.0	https://osf.io/98d5w/
*Design File 3*	100mmbox_Side	Cad File (.FCStd)	CC by 4.0	https://osf.io/3xwf4/
*Design File 4*	250mmBox_Bot	Cad File (.FCStd)	CC by 4.0	https://osf.io/fcjgs/
*Design File 5*	250mmBox_Front	Cad File (.FCStd)	CC by 4.0	https://osf.io/h3juy/
*Design File 6*	250mmBox_Side	Cad File (.FCStd)	CC by 4.0	https://osf.io/s7d6f/
*Design File 7*	250mmBox_Top	Cad File (.FCStd)	CC by 4.0	https://osf.io/smak6/
*Design File 8*	350mm_Base	Cad File (.FCStd)	CC by 4.0	https://osf.io/s3pvu/
*Design File 9*	Frame_Assembly	Cad File (.FCStd)	CC by 4.0	https://osf.io/f8uj2/
*Design File 10*	FoamPiFrameBuild	Assembly Figures (.pdf)	CC by 4.0	https://osf.io/b5rkq/
*Design File 11*	SuppFigureA1_BoxinBox	Figures (.png)	CC by 4.0	https://osf.io/9ugqz/
*Script File 1*	FoamPi	Python Script (.py)	GNU GPLv3	https://osf.io/d5g3m/
*Script File 2*	TempHeightCorrect	Python Script (.py)	GNU GPLv3	https://osf.io/6kz39/
*Script File 3*	SummaryT_H_m	Python Script (.py)	GNU GPLv3	https://osf.io/pmd9j/

Design File 1–8 are the files for each of the sides of the two (inner and outer) reaction boxes which can either be laser cut or cut and drilled by hand using the files as stencils. Design file 9 is the fully assembled frame including the time of flight laser stand and the connection of the force bar to the base and underside of the outer reaction box. Design file 10 is a supplementary figure showing the cardboard box in box design. Script File 1 is the python script installed on the RPi to run the FoamPi and log temperature, height and mass data. Script File 2 corrects raw time–temperature data to adiabatic temperature rise, and corrects and smooths the temperature, height and mass data and generates a corrected.csv file. Script File 3 extracts important points from a batch of corrected log files.

## Bill of materials summary

Full bill of materials required for building the FoamPi also available in the online repository.

**Table:** 

**Designator**	**Component**	**Number**	**Cost per unit /GBP**	**Total cost /GBP**	**Source of materials**	**Material type**
**Electronics**						
RPi	Raspberry Pi 4 starter kit	1	79.02	79.02	RS components	Other
RPi Screen	Raspberry Pi LCD touch screen	1	58.22	58.22	RS Components	Other
RPi Keyboard	RPi Keyboard	1	16.08	16.08	RS Components	Other
GrovePi+	Seed Studrio GrovePi + Bridge	1	33.60	33.60	RS Components	Other
MCP9600	Seeed Thermocouple Amplifier	1	13.81	13.81	RS Components	Other
HX711	Seeed HX711 Board	1	4.00	4.00	Cool Components	Other
VL53L0X	Seeed TOF Board	1	11.70	11.70	RS Components	Other
Thermocouple	Type K Thermocouple	1	3.46	3.46	RS Components	Other
Load Cell	Weight Sensor (0 – 3 kg)	1	6.98	6.98	Cool Components	Other
**Reaction Box and Supports**						
Out-S	6 mm Birch (250 × 256 mm)	2	5.98	15.07	Cutmyplastic	Wood
Out-FB	6 mm Birch (262 × 256 mm)	2	6.28	12.58	Cutmyplastic	Wood
Out-Bot	6 mm Birch (250 × 250 mm)	1	2.92	5.84	Cutmyplastic	Wood
Out-Top	6 mm Birch (262 × 262 mm)	1	2.92	2.92	Cutmyplastic	Wood
In-S	6 mm Birch (100 × 106 mm)	2	4	8	Cutmyplastic	Wood
In-FB	6 mm Birch (106 × 112 mm)	2	4	8	Cutmyplastic	Wood
In-Bot	6 mm Birch (100 × 100 mm)	1	2	2	Cutmyplastic	Wood
Base	6 mm Birch (350 × 350 mm)	1	4.91	4.92	Cutmyplastic	Wood
FoamFiller	Polyurethane Gap filler	1	7.54	7.54	RS Components	Polymer
KTube	RS Pro Clear Tube 1 m × 12 mm	1	9.01	9.01	RS Components	Polymer
Strut	RS Pro Strut 20 × 20 mm	1	7.54	7.54	RS Components	Metal
StrutL	RS PRO Connector Bracket	1	8.34	7.34	RS Components	Metal
FixStrut	RS Pro Fixing Component	1	10.66	10.66	RS Components	Metal
M4Bolt	RS Pro M4x10mm	1	8.08	8.08	RS Components	Metal
Feet	Feet for Base	2	1.99	3.98	Screwfix	Other
BaseBolt	M6 × 25 mm 50 Pack	1	4.39	4.39	Screwfix	Metal
M6Hex	Easyfix A2 SS Hex Nut	1	6.19	6.19	Screwfix	Metal

Total Cost: GBP 350.93.

## Build instructions

### Reaction box and stand build

**Fig. 2:**
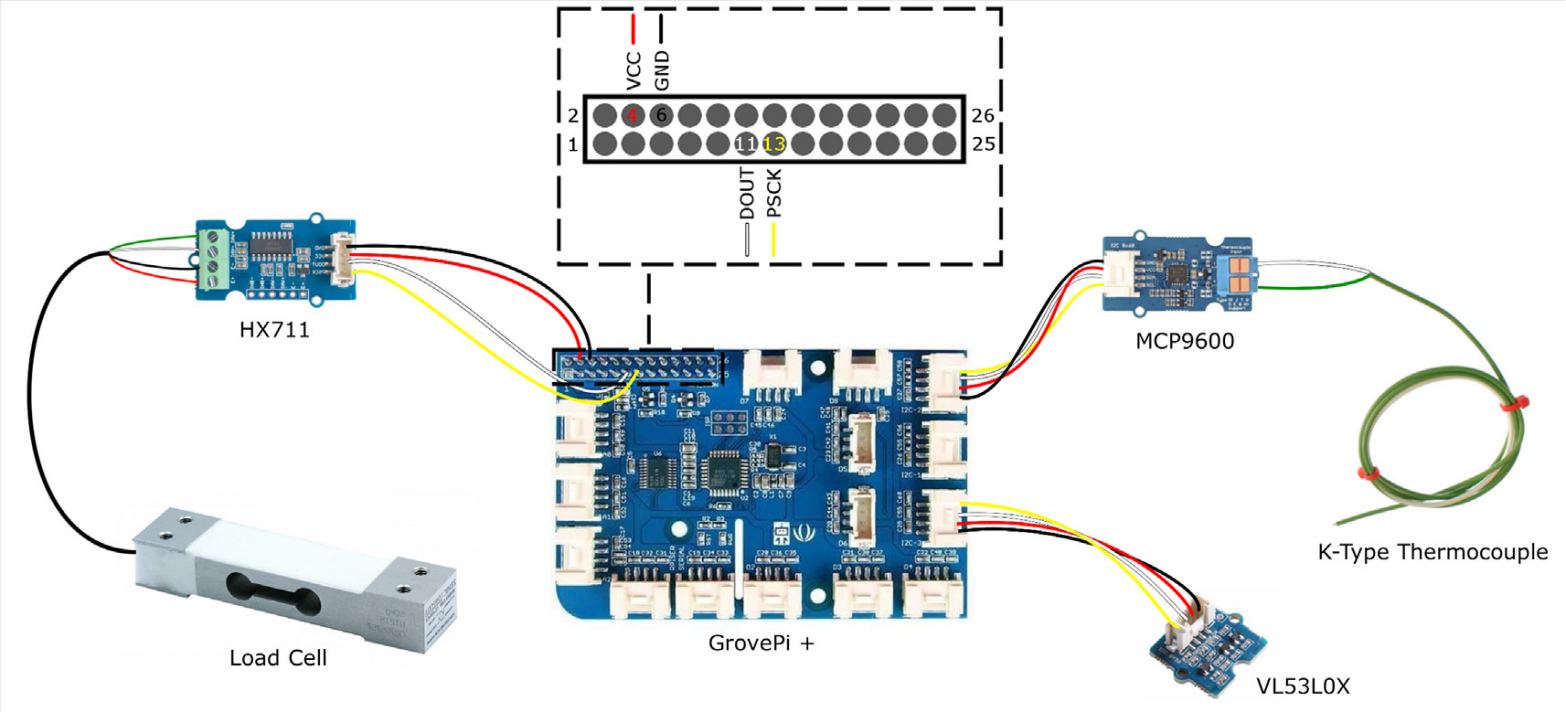
Wiring diagram for the RPi and sensors for the FoamPi, MCP9600 and VLX53L0X are connected via GrovePi I2C ports and HX711 is connected via RPi GPIO pins as shown in the insert.

**Fig. 3:**
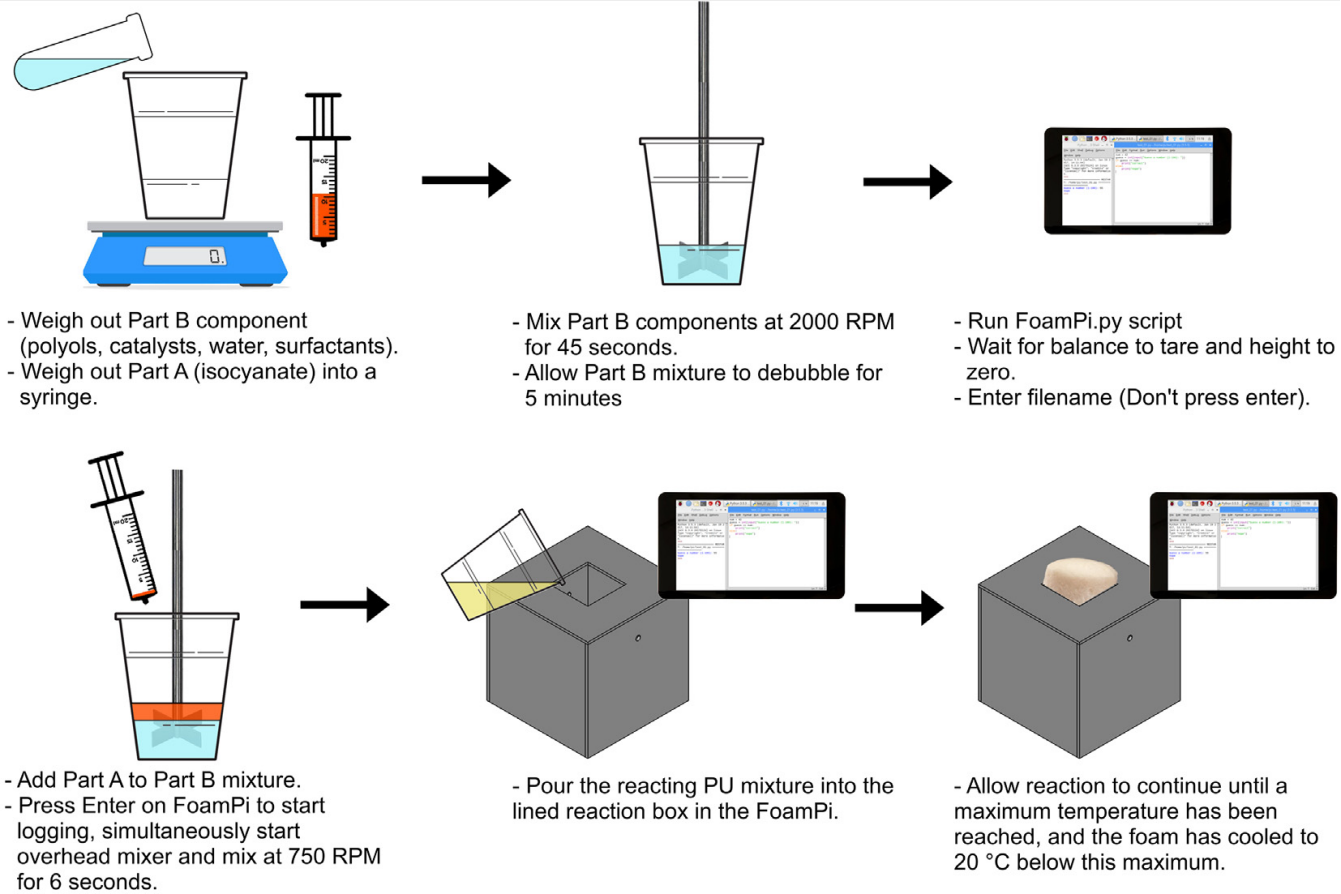
The workflow for running a polyurethane reaction in the FoamPi.

A more detailed build for the reaction box and FoamPi frame build is given in Design File 10. The wood for the reaction boxes can be laser cut using the design files 1–8 or cut by hand and drilled as required using the design files as templates. The 100 mm box is assembled as shown in Design File 10: Figure 1 and is glued and clamped together. The same assembly is used for the 250 mm box as shown in Design File 10: Figure 1. The Load Cell is bolted to the underside of the outer 250 mm box using the M6 bolt (BaseBolt) using the two central mounting holes and is secured using one H6Hex nut per screw and to the Base 350 mm board as shown in Design File 10: Figure 2. Feet are mounted to the four corners of the base board. The KTube is then used to position the 100 mm reaction box inside the larger 250 mm box as shown in Design File 10: Figure 3. FoamFiller is sprayed in the space between the two boxes to insulate the void between the two boxes. Once the foam has cured the top of the 250 mm box can be glued in place.

**Fig. 4:**
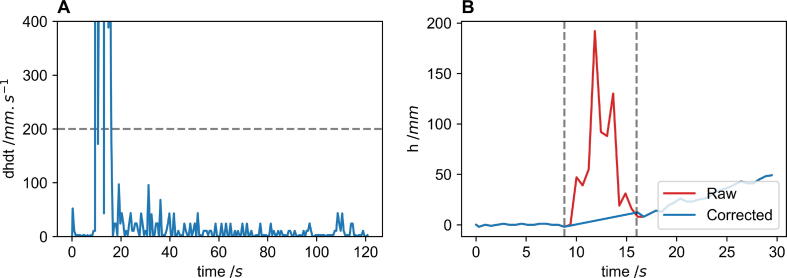
(A) The derivative of height data with respect to time in blue with the grey dashed line indicating the threshold for removing erroneous height data. (B) The height data within the region identified in the derivative of the height data, showing the raw erroneous data in red and the linear fit used to correct the data between the two grey dashed lines. (For interpretation of the references to colour in this figure legend, the reader is referred to the web version of this article.)

**Fig. 5:**
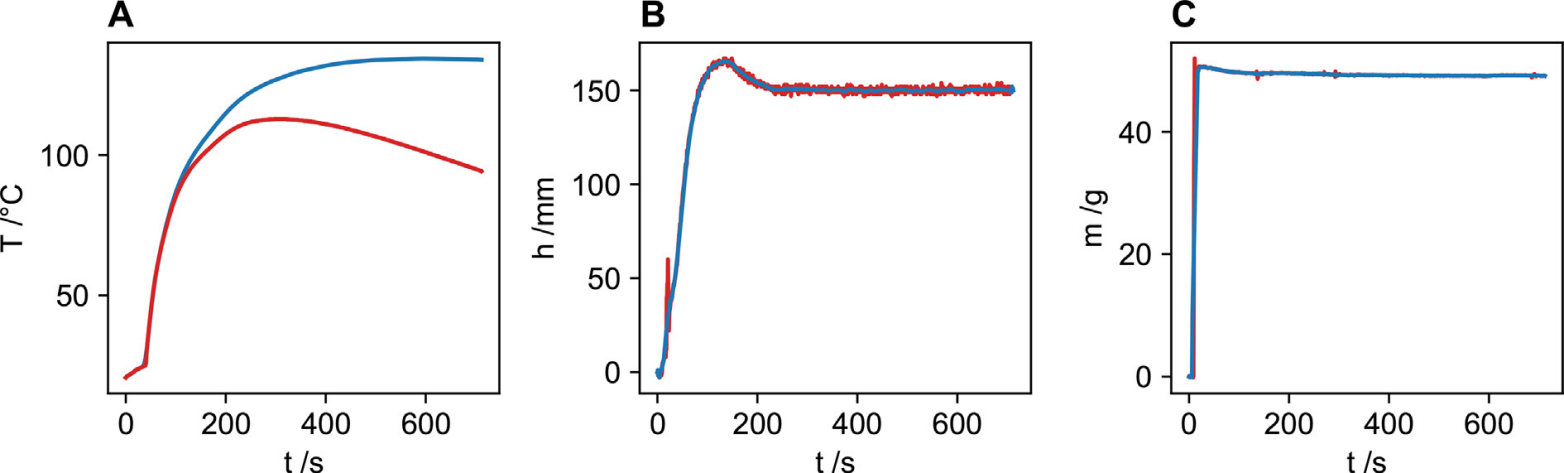
Data captured directly by the FoamPi is shown in red, and the traces that have been corrected using the script TempHeightCorrect.py are shown in blue. The temperature rise trace (A) has had an temperature correction such that the trace shows that for adiabatic temperature rise, whilst the height trace (B) and mass trace (C) have been corrected for erroneous data and have had a 21 point moving average applied to smooth out noise. (For interpretation of the references to colour in this figure legend, the reader is referred to the web version of this article.)

The stand for the TOF laser framework is assembled next. The strut is cut into three lengths, for the base (100 mm), upright (380 mm) and arm (180 mm). These are mounted as shown in Design File 10: Figure 4 using the connector bracket (StrutL) and fixing component (FixStrut) and M4 bolts (M4Bolt). The stand is mounted to the 350 mm baseboard (BaseBoard) using the same fixing components (FixStrut and M4Bolt). The final assembly of the frame for the FoamPi is shown in Design File 10: Figure 5.

### Rpi hardware and software setup

The RPi screen is wired and setup according to the screen’s official documentation (available here). A pre-installed “NOOBS” micro SD card was used in the RPI. The Grovepi + bridge was setup according to the official Grovepi documentation (available here). The temperature sensor (MCP9600) was connected to I2C-1 port and the time of flight board (VL53L0X) was connected to I2C-3 port. The load cell board (HX711) was wired directly to the GPIO pins on the Grovepi + bridge (VCC − 4, GND − 6, DOUT − 11 and PSCK − 13). The wiring diagram is shown in Fig. 2.

The FoamPi uses a Python script for logging and correcting data and several Python packages are required for this script. Specifically packages for the three sensors are required and these are given in Table 1.

**Table 1:** Python Packages required for the FoamPi.

Sensor	Github Repository
HX711	https://github.com/tatobari/hx711py
MCP9600	https://github.com/pimoroni/mcp9600-python
VL53L0X	https://github.com/pimoroni/VL53L0X-python

## Operation instructions

The reaction box needs to be lined to ensure no polyurethane foam adhesion to the wood surfaces; waxed baking paper works well and is reusable between runs. The thermocouple is centred in the reaction box to ensure maximum insulation by the reacting foam (this is done by eye). Once the reaction box is prepared for the reaction reagents can be weighed out and pre mixed. Part B (all reagents except the isocyanate) are weighed out and isocyanate (part A) is weighed out separately in a syringe. Part B is mixed using an overhead mixer at 2000 RPM for 45 s (this can be varied as appropriate for the mixing blade/ foaming system) Part B is then left to debubble for 5 min before initiating the reaction. Fig. 3 shows the workflow for running a polyurethane reaction and monitoring the reaction with the FoamPi. This workflow is adapted from the industry handbook by The Dow Chemical Company [14].

Boot the FoamPi, open, and execute the script FoamPi.py in your choice IDE. On running the script it will tare the balance, zero the height measurement and then prompt for a filename. Pressing enter after entering the filename starts logging data, so it is suggested to type in the filename and wait until reagent mixing starts before pressing enter to synchronise the reaction and logging time. Mix the Part A and Part B polyurethane at 750 RPM for 6 s with an overhead mixer (this can be varied as appropriate for the users foaming conditions) and press enter to start logging simultaneously Once mixing is complete, transfer the reacting contents into the lined reaction box. The FoamPi.py script will display the time/temperature/height and mass data live. The temperature will rise as the reaction proceeds, reach a maximum and start decreasing; this information can be used to determine the end of the reaction. The end point can be determined by waiting for the temperature to drop 20 °C below the maximum temperature reached during the reaction. This ensures sufficient cooling data to do an adiabatic temperature rise correction.

Adiabatic temperature rise correction, error correction and noise reduction are done using the script TempHeightCorrect.py that corrects all the files in a folder. The script will prompt you to select the working directory. Once the script has executed it will generate a new.csv file for each of the reaction run files with the naming convention of “filename_corrected.csv”. The final script SummaryT_H_m.py file extracts important kinetic points from the corrected files. This runs the same as the correction script and will again prompt you to select a folder which contains the corrected data. This script runs though all “.csv” files so ensure that you separate corrected and uncorrected data. Once executed the script generates a file, “adiabatic temperature risesumm.csv” containing the extracted points. These include: maximum temperature difference (ΔT_max_), time of maximum temperature (t_Tmax_), max height (h_max_), time of max height (t_hmax_), final mass (m_fin_), mass loss (Δm) and sag (Δh_sag_).

The corrected adiabatic temperature rise data as well as extracted points can be used for kinetic and foam formulation work including, but not limited to, determining isocyanate conversion, gelling and blowing rates, determining catalyst/ surfactant activities or gaining an understanding of expected cell morphological features.

## Validation and characterization

### Data correction

The script TempHeightCorrect.py is used to correct the raw data captured by the FoamPi. Temperature data needs a heat loss correction to translate the raw temperature–time data into adiabatic temperature rise data that can be used to determine isocyanate conversion during the reaction. This heat loss correction follows that used by Lipshitz and Macoscko, 1977 [1] using the energy balance shown in Eq. (1).

(1) $$\rho c_{p}\frac{dT_{\exp}}{\mathrm{dt}}\;=\;\left(-\Delta H\right)k_{1}\;-\;U\left(T_{\exp}\;-\;T_{\mathrm{amb}}\right)$$

where ρ is the density (kg.m^−3^), c_p_ is the heat capacity (J.g^−1^_._ K^−1^), T_exp_ is the reaction temperature (K), t is the time (s), ΔH is the heat of formation of products (J.mol^−1^), k_1_ is the rate of formation of products (mol.m^−3^.s^−1^), U is the global heat transfer coefficient (J.K^−1^.m^−3^.s^−1^) and T_amb_ is the ambient temperature (K). When no reaction occurs (a long time into the reaction, when only cooling due to heat loss occurs), Eq. (1) can be simplified and integrated between t and t_0_ to yield Eq. (2).

(2) $$\text{ln}{\text{(T}}_{\text{exp}}\;\text{-}\;{\text{T}}_{\text{amb}}\text{) = ln}{\text{(T}}_{\text{0}}\;\text{-}\;{\text{T}}_{\text{amb}}\text{) - U'(t -}\;{\text{t}}_{\text{0}}\text{)}$$

Eq. (2) can then be plotted and the slope during the period of cooling will give us the overall heat transfer coefficient U' (U/ρc_p_). The experimental temperature can then be adjusted for heat loss by combining Eq. (3), the equation for an adiabatic chemical reaction with Eq. (1) and integrating to yield Eq. (4),

(3) $$\rho c_{p}\frac{dT_{\mathrm{ad}}}{\mathrm{dt}}=\;\left(-\Delta H\right)r_{1}\;$$

(4) $${\text{T}}_{\text{ad}}\text{=}\;{\text{T}}_{\text{exp}}\text{+ U'}\int_{\text{0}}^{\text{t}}\text{(}{\text{T}}_{\text{exp}\;}\text{-}\;{\text{T}}_{\text{o}}\text{)}\text{dt}$$

where T_ad_ is the adiabatic temperature corrected for heat loss. The use of these equations and this correction rely on a few important assumptions.

1 The c_p_ of the foams remains constant over the entire temperature range.
2 There are no external sources of heat.
3 The isocyanate only reacts with water and hydroxyls and forms only urea, urethane groups and CO_2_.
4 The solution is well mixed and homogenous.

The use of this heat loss correction, allows for the monitoring of the both fast and slow reactions and means that the reaction vessel does not need to be completely insulated. This heat loss correction also corrects for any heat loss through the top of an open reaction vessel.

Height and mass data are also corrected for any outlier data. A mean and standard deviation method is used to determine outlier points, where the mean is a 21-point moving average and the standard deviation is determined from this subset. Points are classed as outliers if there are outside of 2 × mean ± SD. If this is the case, the point proceeding it replaces the outlying point. A special case is considered for the height data near the start when reagents are being poured into the reaction vessel. There is a chance that the users hand or the reagent mixing vessel may interfere with the laser’s pathway leading to incorrect data. To correct for this derivative of the height data is found, and the first 200 data points are analysed (roughly 125 s). If the value of the derivative is above a threshold (2 0 0) as shown in Fig. 4 (A), data is considered erroneous and in need of correction. To correct for the data a linear regression is done between first erroneous and final erroneous data point and this fit is used to replace erroneous data, as shown in Fig. 4 (B).

Following these corrections, a 21-point moving average is used to smooth noise for the temperature, height and mass data. Fig. 5 shows the shows the raw data in red and the corrected data in blue for a typical polyurethane formulation. Fig. 5 shows that the script TempHeightCorrect.py is able to correct for temperature loss and remove erroneous data from the height and mass curves.

### Precision

**Fig. 6:**
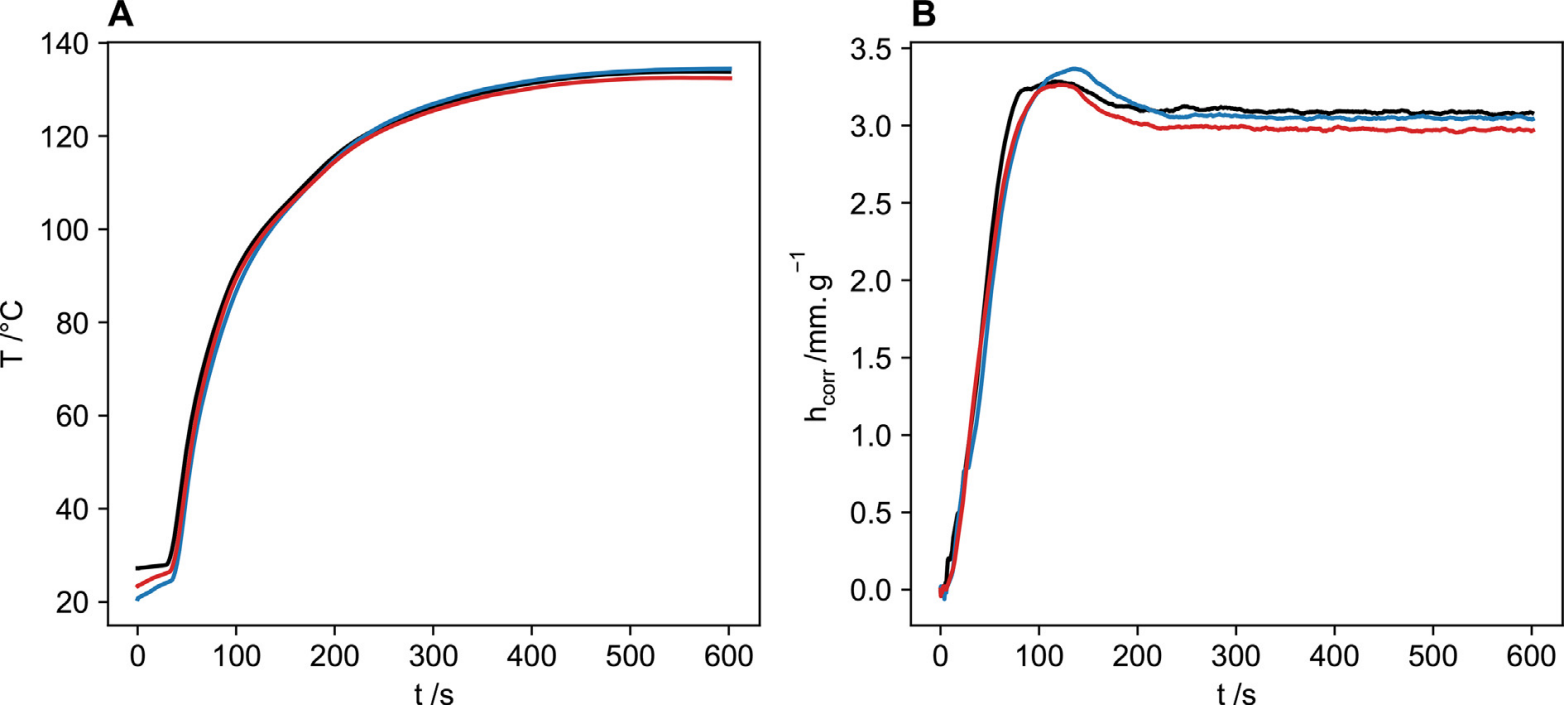
Repeat polyurethane formulation runs logged on the FoamPi. (A) Corrected temperature rise curve and (B) the corrected height curve divided by the final foam mass to normalise the three curves.

A foam formulation was run in triplicate to determine the precision of the FoamPi. Fig. 6 shows the temperature and height traces for these three replicate runs. Height data is standardised by dividing by the final mass of the foam after reaction to normalise for different masses of foams added to the reaction vessel. The three corrected temperature traces are in very good agreement with a mean maximum temperature change of ΔT_max_ = 109.8 °C and a relative standard deviation (RSD) of 3.33 %. The height curves were also in good agreement with the mean maximum height, h_max_ = 3.31 mm.g^−1^ and a RSD of 1.66 %. In addition to this the final heights are also in good agreement with the mean final height, h_fin_ = 3.03 mm.g^−1^ and a RSD of 1.89 %. These results show that the FoamPi has good precision for recording temperature data and if the height data is normalised by dividing by the final foam mass then precision for the height data is also high.

**Fig. 7:**
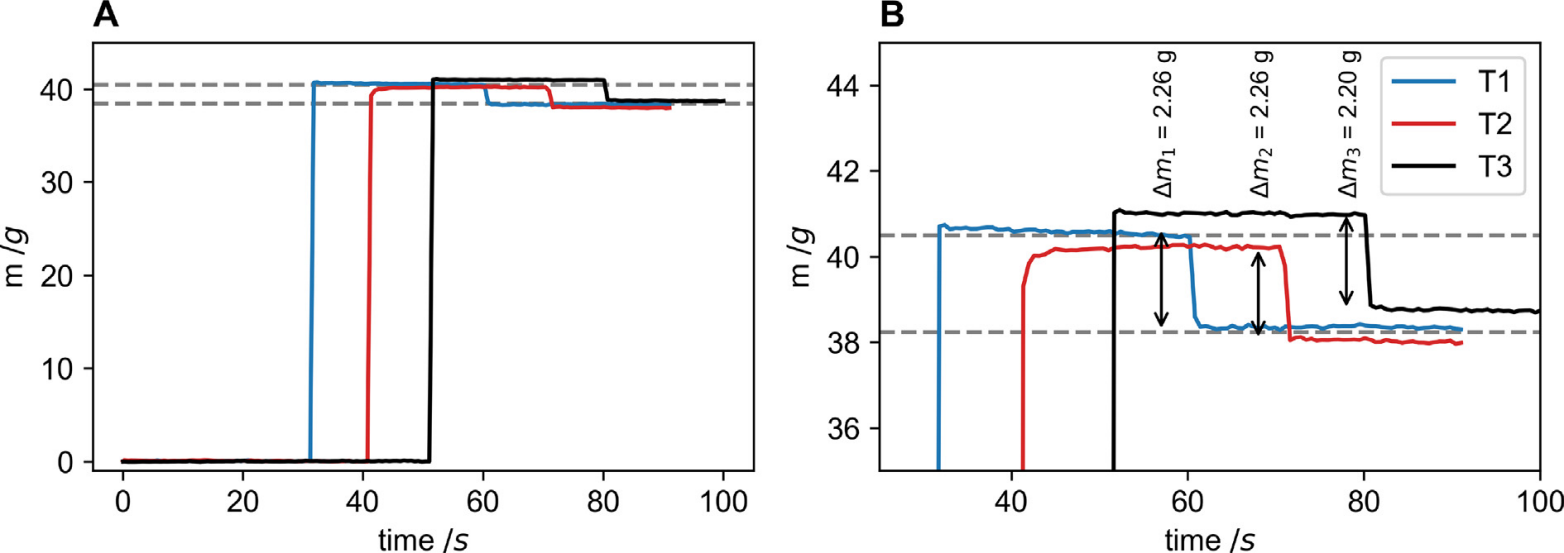
Repeat curves testing the sensitivity and precision of the load cell with (A) showing the addition of the 40.5 g reaction mass and (B) the change in mass after removing a 2.25 g weight from reaction vessel at varying times for three runs. For both figures, the dashed lines show the expected mass before and after the 2.25 g weight has been removed.

The sensitivity of the mass sensor was also tested to determine whether the load cell would be precise enough to determine the mass loss occurring during a foaming reaction when compared to the larger mass of the reaction box and foaming reagents. The FoamPi software was run and after a known time a 40.5 g weight was added to the reaction vessel. This is equivalent to the amount of reagent required to make a polyurethane foam in the reaction vessel. The software logged the mass for a further 30 s upon which a 2.25 g mass was removed from the vessel to imitate the loss of CO_2_ due to cell wall rupturing. This was repeated in triplicate. Fig. 7 (A) shows the mass data for the three runs. The mean error between the expected mass and measured mass was 0.3 g (0.75 %) and the maximum error was 0.45 g (1.1 %). Fig. 7 (B) shows the change in mass (*Δm*) for each test once the 2.25 g mass was removed. The mean error between the expected and measured mass was 0.023 g (1.04 %) and the maximum error was 0.05 g (2.22 %). This shows that even though the mass of reagent and mass of CO_2_ loss is low when compared to the mass of the reaction box, the load cell is still precise enough to accurately determine these changes.

## Limitation of the mass sensor

**Fig. 8:**
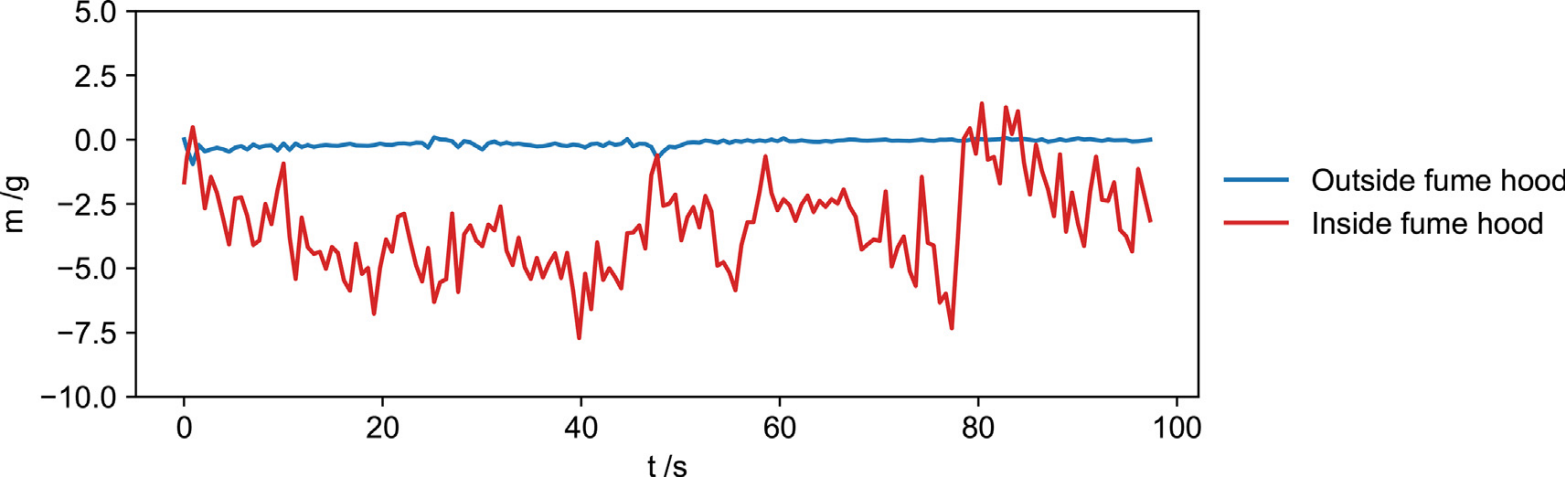
Limitation of the mass sensor when used in a fume hood, airflow causes large variations in mass readings.

One limitation of the FoamPi is the use of the mass sensor inside a fume hood (where due to the nature of the chemicals involved most polyurethane foam reactions take place [15]). Due to the high rate of airflow inside the fume hood, there is a large amount of drift of the mass data. This is shown in Fig. 8 where the mass data is recorded without a load when run both inside and outside of a fume hood. The observed mass changes by up to 7.5 g whilst in the fume hood and considering that the amount of mass lost during these polyurethane reactions is between 1 and 2 g, sensitivity is lost in this environment and the FoamPi is not able to capture meaningful mass data during the reaction. The sensor can be used to determine the final foam mass after the reaction, outside of the fume hood to normalise height data, however real time data is not accurate. This could likely be overcome by placing the FoamPi in a glovebox inside the fume hood, which could be vented once the reaction was complete, however this needs confirmation.

### Surfactant replacement study (case use)

An example case use of the FoamPi is for trialling new surfactants for polyurethane foam formulations. Surfactants play an important role in polyurethane foam; where they increase compatibility between components, help with foam bubble generation and with cell wall stabilisation. The type of surfactant, the chemical structure and the amount used also have large effects on the resulting physical properties and cell morphology of the foam. Surfactants can affect the cell size, ratio of open to closed cells and density that results in very different physical and mechanical properties. Surfactant and foam manufacturers will regularly trial new surfactants or formulations and an early indicator of final physical properties can be advantageous to determining suitability of a new surfactant.

In this case use two methylene diphenyl diisocyanate (MDI) based flexible foam formulations are run on the FoamPi. Formulations for the two samples are identical with only the surfactant changing to emulate the trialling of a new surfactant. Samples are designated as OC (old surfactant) and CC (new surfactant).

**Fig. 9:**
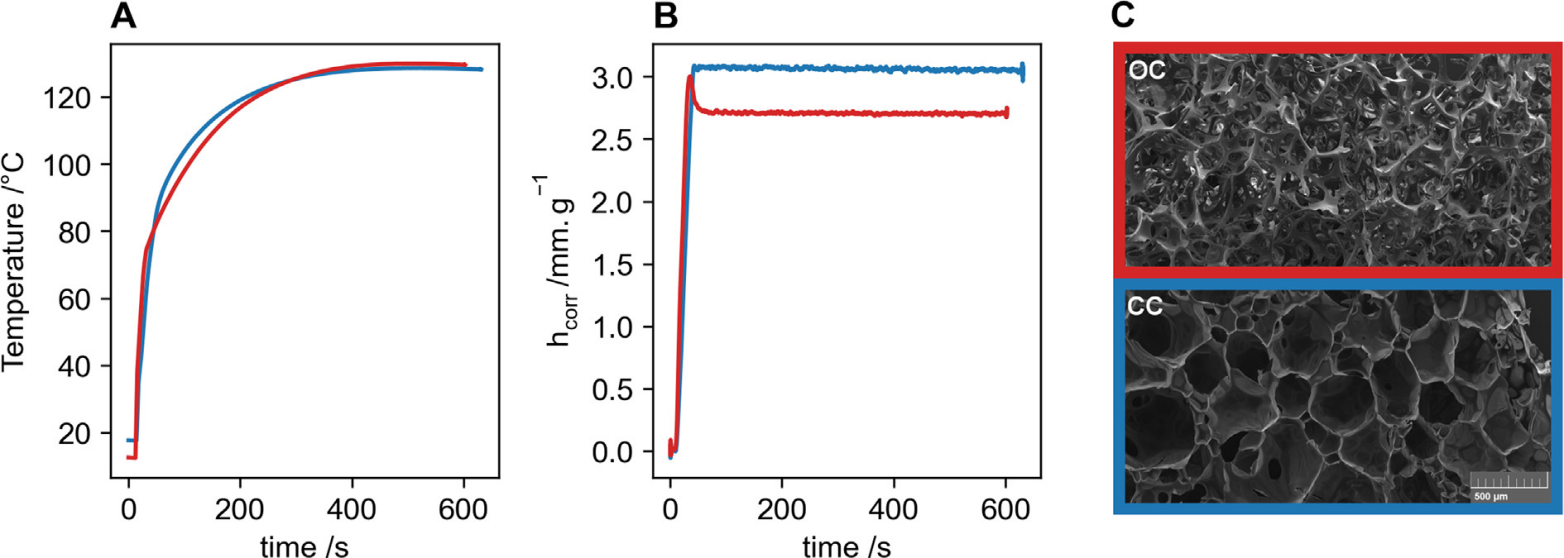
A use case for the FoamPi shows two different surfactant formulations, one that leads to closed cells (CC, blue) and one that leads to open cells (OC, red). Temperature curves (A) show little difference between the two formulations, the height curve (B) shows that they rise to similar heights but OC (red) sags, indicating open cells. This is confirmed in the micrographs (C) with OC showing fully open cells and CS showing closed cells. (For interpretation of the references to colour in this figure legend, the reader is referred to the web version of this article.)

The two samples were run on the FoamPi and corrections were done using the associated scripts. The temperature and height traces are shown in Fig. 9. The temperature traces are near identical for the two samples, OC in red and CC in blue, this is expected as changing the surfactant should not alter the kinetics of the foaming reactions. The height traces are very different with both samples rising to the same height, however the OC sample then quickly “relaxes” or “sags” to a lower final height. This process of sagging is an obvious indicator of open cells. The CC sample shows no sagging and remains at the maximum height. It would be possible at this point to assume that this new surfactant would not be a suitable replacement for the current one, as the complete lack of sagging suggests, few to no open cells. This change in cell morphology would have a considerable impact on the foams physical properties. Scanning electron microscopy (SEM) was done to confirm this is the case and Fig. 9 (C) shows the micrograph of OC (red), which is completely open cell and the micrograph for CC (blue) where all cell walls are still visible and the foam consists of closed cells. It is the combination of temperature and height data that allows this insight into the properties of the foam. If the only data available was the temperature rise curves then the difference in these two surfactants would not be apparent.

### Summary of validation and characterisation

• The FoamPi is able to successfully correct for heat loss, allowing for data to be treated as adiabatic.
• Raw height and mass data is smoothed using a 21 point moving average and is able to remove erroneous height data due to pouring of reagents into the reaction vessel.
• The FoamPi has good reproducibility with the maximum temperature change having a RSD = 3.33 %, the maximum height had a RSD = 1.66 % and the final height had a RSD = 1.89 %.
• Even though the mass of reagents and mass loss is low in comparison to the mass of the reaction vessel the load cell has good sensitivity with the maximum error between expected reagent mass and actual reagent mass equal to 1.11 %. Similarly the maximum mass loss error was low at 2.22 %.
• The load cell shows a limitation when used in a fume hood as the airflow causes too much drift in the values.
• A case use for the replacement of a surfactant in a PUF formulation is demonstrated and use of the FoamPi immediately indicates that the replacement surfactant is not a suitable.

## Conclusions

A low cost Raspberry Pi based apparatus for monitoring the kinetics and rise profile of polyurethane foams has been developed and tested. The apparatus physical structure is made from easily accessible and low cost materials that only require laser cutting (or manual cutting). The electronics are based on a Raspberry Pi with GrovePi + bridge to simplify the wiring process. Three sensors (MCP9600, HX711 and VL53L0X) were found to be suitable to log temperature, height, and mass respectfully. A second simplified system without mass sensor, GrovePI + and touchscreen, which could be run in a headerless configuration, was also described to save further on costs. A script to remove erroneous data, correct temperature for heat loss and smooth data was developed and validated. The FoamPi was tested with repeated formulations and the system was shown to have good precision. Whilst the temperature and height sensor had good sensitivity in all environments the mass sensor showed large amounts of drift when used in a fume hood, a limitation of the system. Finally a case use was demonstrated where the FoamPi could identify a foam with open or closed cells from the height rise data.

## CRediT authorship contribution statement

**Harry C. Wright:** Conceptualization, Data curation, Writing – original draft, Visualization, Investigation. **Duncan D. Cameron:** Supervision, Writing – review & editing. **Anthony J. Ryan:** Supervision, Writing – review & editing.

## Declaration of Competing Interest

The authors declare that they have no known competing financial interests or personal relationships that could have appeared to influence the work reported in this paper.
